# Validity of ultrasound imaging for intrinsic foot muscle cross-sectional area measurements demonstrated by strong agreement with MRI

**DOI:** 10.1186/s12891-022-05090-6

**Published:** 2022-02-14

**Authors:** Dallin C Swanson, Joshua K Sponbeck, Derek A Swanson, Conner D Stevens, Steven P. Allen, Ulrike H. Mitchell, James D. George, Aaron Wayne Johnson

**Affiliations:** 1 Department of Exercise Sciences, Brigham Young University, 84602 Provo, UT USA; 2 Electrical and Computer Engineering, Ira A. Fulton College of Engineering, Brigham Young University, Provo, UT USA

**Keywords:** Diagnostic imaging, Foot muscle size, Validity

## Abstract

**Purpose:**

Intrinsic foot muscles maintain foot structural integrity and contribute to functional movement, posture and balance. Thus, assessing intrinsic foot muscle size and strength are important. Magnetic resonance imaging (MRI) has been shown to accurately image the individual muscles but is costly and time consuming. Ultrasound (US) imaging may provide an alternative that is less costly and more readily available. The purpose of this study was to investigate the validity and intratester reliability of US imaging in measuring intrinsic foot muscle size in comparison to MRI.

**Methods:**

US and MRI were employed to measure the intrinsic foot muscle size involving 35 participants (females = 13; males = 22). The scanned intrinsic foot muscles included the flexor hallucis brevis (FHB), abductor hallucis (ABDH), flexor digitorum brevis (FDB), quadratus plantae (QP) and abductor digiti minimi (ADM). Pearson product correlation (r), intraclass correlation coefficients (ICC), standard error of the measurement (SEm) and minimal detectable difference (MDD) were calculated.

**Results:**

High correlations were detected between the US and MRI cross-sectional area (CSA) measurements (*r* = .971 to 0.995). Test reliability was excellent for both MRI and US (ICC = 0.994 to 0.999). Limits of agreement between MRI and US measurements from ranged from 5.7 to 12.2% of muscle size. SEm values for US ranged from 0.026 to 0.044 cm2, while the SEm for MRI ranged from 0.018 to 0.023 cm2. MDD values for US ranged from 0.073 to 0.122 cm2, while MRI ranged from 0.045 to 0.064 cm2.

**Conclusions:**

US appears to be a valid and reliable alternative to MRI when measuring intrinsic foot muscle CSA. While US is less costly and more readily available, the MRI results were shown to be slightly more precise.

## Introduction

Healthy intrinsic foot muscles maintain the structural integrity of the foot [1], are active during functional movement [2], support proper postural alignment [3] and balance control [4]. Poor intrinsic foot muscle strength is associated with excessive pronation [5] and pes planus [6]. Both excessive pronation and pes planus are associated with several common musculoskeletal overuse injuries and conditions including fatigue, increased navicular drop [7], osteoarthritis [8] and running-related injuries [9]. Intrinsic foot muscle atrophy is also related to medical conditions such as Charcot-Marie-Tooth disease (motor and sensory neuropathy), claw toe and hammer toe deformities [10], diabetic neuropathy [11], hallux valgus, pes planus [12], and plantar fasciitis [13]. Thus, the ability to accurately and efficiently measure the intrinsic foot muscle size and strength is important in a clinical setting.

Unfortunately, it is inherently challenging to measure the size and strength of the intrinsic foot muscles due to their small size and depth within the foot’s tissue structure [12]. In addition, these muscles share similar functions, such as maintaining the medial longitudinal arch [14], which makes identifying the specific functional contribution of each intrinsic muscle difficult [10].

Currently, measuring intrinsic foot muscle strength is done using both direct and indirect methods [15]. Direct methods are traditional strength diagnostic tests such as paper grip test, plantar pressure, and handheld dynamometry [10, 16]. Direct methods measure the combined strength of all intrinsic foot muscles working at the same time but are unable to measure the strength of one specific muscle.

Indirect techniques commonly use the muscle’s cross-sectional area (CSA) to estimate intrinsic foot muscle strength [10, 17] Muscle CSA and force production have been shown to be highly correlated in trunk and hand muscles [15, 18] as well as moderately to strongly correlated in intrinsic foot muscles [17, 19]. This relationship is regularly used to estimate muscle strength [18, 20, 21]. Indirect techniques can also isolate single muscles [22] and assess muscle size changes during disease states [19] and across exercise training programs [23].

Magnetic resonance imaging (MRI) and ultrasound (US) imaging are both used to indirectly measure intrinsic foot muscle size and estimate muscle strength [24–27]. MRI is considered the criterion standard due to its high resolution and multi-planar view [10]. MRI is also effective at clearly displaying muscle anatomy by using tissue contrast [28, 29]. Although MRI has the great advantage of being able to accurately image individual muscles, it has the disadvantage of being costly and time consuming. In addition, MRI cannot capture real-time muscle contractions while US is able to perform this dynamic imaging (ref Jon Jacobson book). Thus, US imaging measurements may provide a viable alternative that is less costly and more readily available.

Previous research confirms US yields valid CSA measurements as compared to MRI when measuring larger muscles, such as the trapezius [30] tibialis anterior [31] and rectus femoris [32]. To date, however, no study has determined the accuracy and validity of US measurements involving the relatively small intrinsic foot muscles.

Consequently, this study’s main purpose was to determine whether US can accurately measure the CSA of five intrinsic foot muscles as compared to MRI. A secondary purpose was to reaffirm the reliability of the US and MRI measurements as shown in another study [26]. Our research hypothesis was that the US method can accurately quantify the CSA of five different intrinsic foot muscles and correlate highly with the MRI method.

## Methods

Thirty-seven participants were recruited for this study, and 35 participants completed all study requirements (female: *n* = 13; mean age ± SD = 25.4 ± 6.8 years; mean height ± SD = 180.7 ± 7.0 cm, body mass ± SD = 82.3 ± 8.9 kg; male: *n* = 22, mean age ± SD = 23.2 ± 4.6 years, mean height ± SD = 168.2 ± 5.3 cm, mean body mass ± SD = 68.7 ± 11.2 kg). Participants were required to be 18 years or older and free from any lower extremity injury within the previous one month or any leg/foot surgery within the previous year. Participants were from the university community including students, staff and faculty. In addition, participants who could not safely receive the MRI scan due to the presence of ferrous-magnetic metal objects within the body, fresh tattoos, a pacemaker, or an implantable cardioverter defibrillator were excluded. Two participants were dropped from the study because of these exclusion criteria.

Each participant provided informed consent by reading, asking relevant questions, and signing the informed consent form approved by the University’s Institutional Review Board (Brigham Young University’s Institutional Review Board of the Human Research Protection Program, study protocol, IRB2019-375). This Institutional Review Board approved all experimental protocols. All methods were carried out in accordance with relevant guidelines and regulations (Federal regulation: 45 CFR 46.111). Each participant completed a safety screen before any MRI testing. Each participant attended one MRI session and one US session. The testing session order was randomized, with each session completed within an hour of one another.

### Imaging preparation

**Fig. 1:**
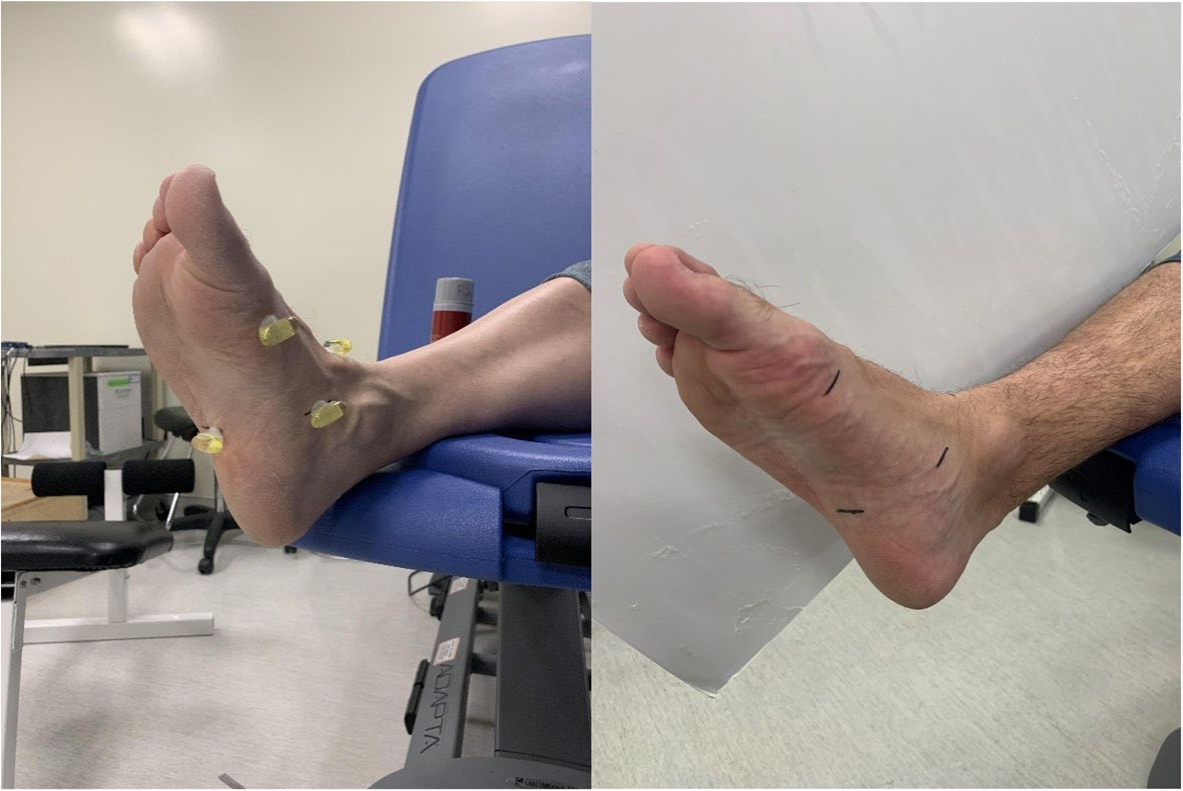
Markings made on the foot for US imaging and MRI fish oil capsule placement

Muscle imaging included scanning the flexor hallucis brevis (FHB), abductor hallucis (ABDH), flexor digitorum brevis (FDB), quadratus plantae (QP), and abductor digiti minimi (ADM). To identify the FHB muscle, a reference mark was made proximal to the head of the first metatarsal, at 10% of the truncated foot length [33]. To image the other muscles, four reference marks were made perpendicular to the longitudinal axis of the foot’s medial, lateral, dorsal, and plantar surface. The medial reference mark was positioned on the navicular tuberosity for both the MRI and US imaging. The other three reference marks were made in the coronal plane transversely across the foot in alignment with this medial mark. Each mark was made on the skin using a marker (Fig. 1). Thus, four muscles were measured in line with the navicular tuberosity and one muscle was measured at 10% of the truncated foot length. All muscles were measured using the short axis (coronal) image for MRI and US to provide two-dimensional data.

### MRI scans

A 3 Tesla magnet (TIM-Trio 3.0T MRI, Siemens, Erlangen, Germany) was employed using a 3-dimensional spoiled gradient echo sequence prescribed with the through-plane direction perpendicular to the long axis of the entire foot. Slice thickness was 1mm, slices were contiguous with no gap or overlap between them. Scan parameters were: TE/TR = 10.8/4.9 ms; matrix size: 416 × 300 × 288 pixels; resolution: 0.4 × 0.4 × 1 mm; Field of view: 150 × 108 × 288 mm; flip angle: 15 degrees; acceleration factor: 2; bandwidth: 130 Hz/pixel; total acquisition time: 4:42.

Participants completed an MRI safety screening before entering the magnet room. Before testing, fish oil capsules (Member’s Mark, Sam’s West Inc., Bentonville, Arkansas) were attached to the participant’s skin via double sided Velcro over each reference mark. The capsules were positioned with the long axis parallel to the coronal plane (Fig. 1) and were visible on the MRI images. Each capsule served as a fiducial marker so each MRI scan could be taken at the correct location. The right foot was scanned first. To do so, it was placed in an 8-channel foot/ankle coil (ScanMed, Omaha, NE, USA). The left leg was scanned second, each foot was scanned once. Light sandbags and wedges were employed to minimize foot movement during imaging.

**Fig. 2:**
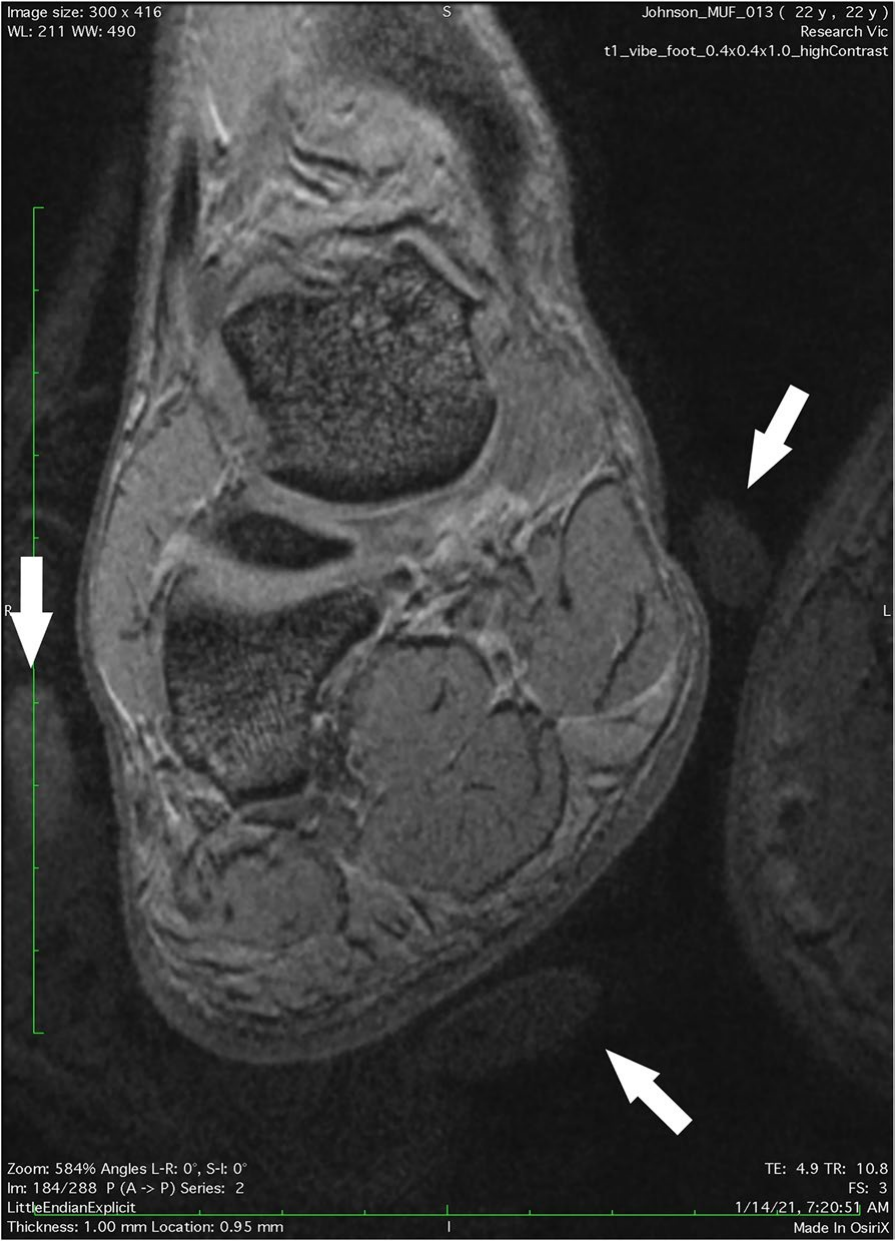
Location of fish oil capsules in MRI scan

The MRI data were captured as a large-block and formatted to appear as multiple images. The image that best intersected the center of the reference fish oil capsules was selected for data analysis (Fig. 2). Due to the 1mm slice thickness the center of the fish oil capsules on MRI could only be identified with a precision of ± 0.5mm.

### US imaging

Participants were seated in a comfortable back-supported position on a treatment table. During imaging, the participant’s hip joint was externally rotated slightly to allow access to the foot’s plantar surface. A bolster was placed underneath the participant’s knee, with the ankle set at approximately zero degrees dorsiflexion throughout imaging.

All images were taken by the same imager who has two years of scanning experience with specialized training of intrinsic foot muscle imaging. Each US muscle image was collected using a ML6-15‐D matrix linear transducer probe (LOGIQ S8; GE Healthcare, Chicago, IL). Settings were set to optimize image quality. Scanning depth (3 cm), frequency (8 MHz), focal position, and time‐gain‐compensation were kept constant.

**Fig. 3:**
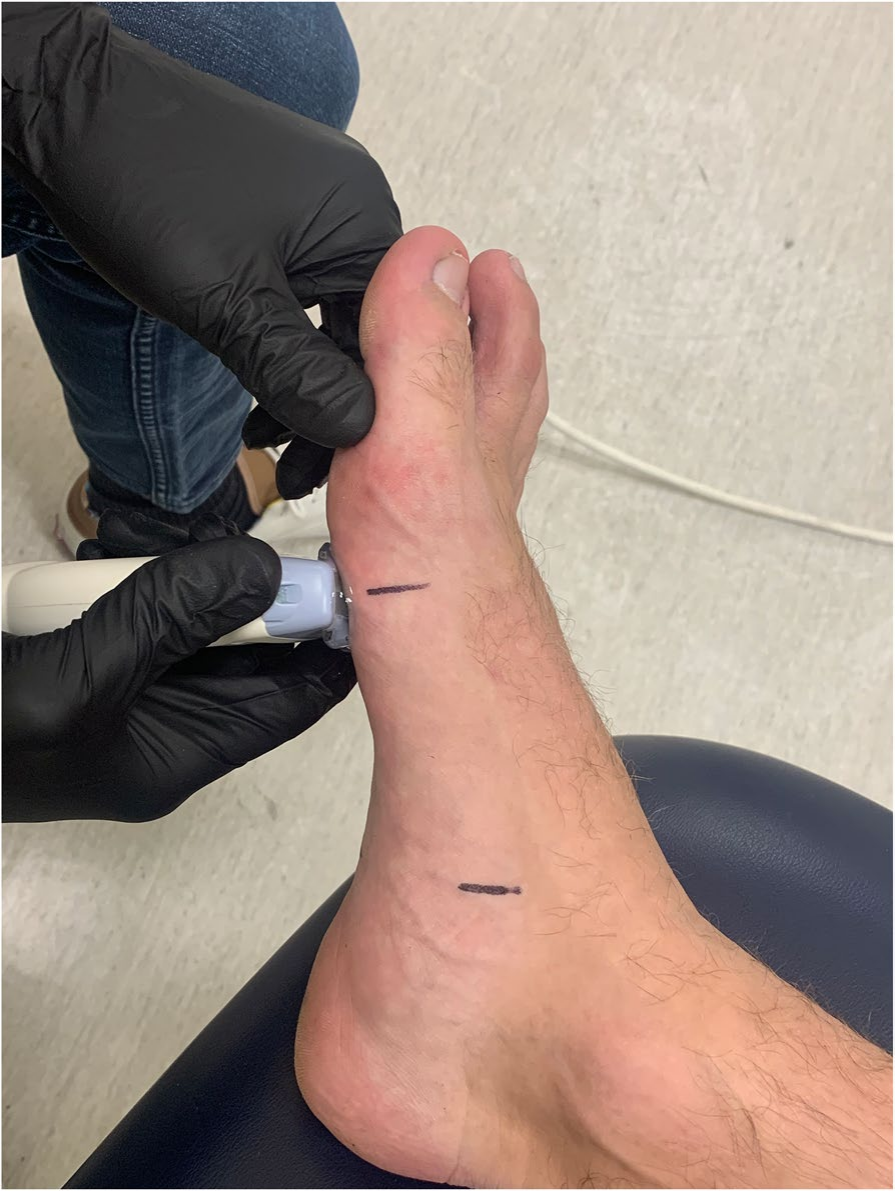
Location of US transducer probe placement for the flexor hallucis brevis muscle

For each US CSA measurement, two separate cine loops were recorded per muscle per foot with the transducer probe removed from the participant’s foot and repositioned between cine loop recordings. The transducer probe was placed transversely to the long axis of the foot to obtain a 2-dimensional slice within the coronal plane on the medial-plantar side of the foot over each corresponding mark (Fig. 3). For the FHB, the muscle body was first found by locating the flexor hallucis longus tendon, sesamoid bones and first metatarsal head. The probe was then moved proximally over the FHB reference mark. The ABDH muscle was imaged at the medial midfoot reference mark using the navicular tuberosity. For the FDB, and QP the transducer probe was positioned on the foot’s plantar midfoot surface and aligned with the reference marks. The ADM muscle was imaged using the same short axis plane as the ABDH, FDB and QP with the transducer probe positioned at the lateral-plantar midfoot reference mark.

**Fig. 4:**
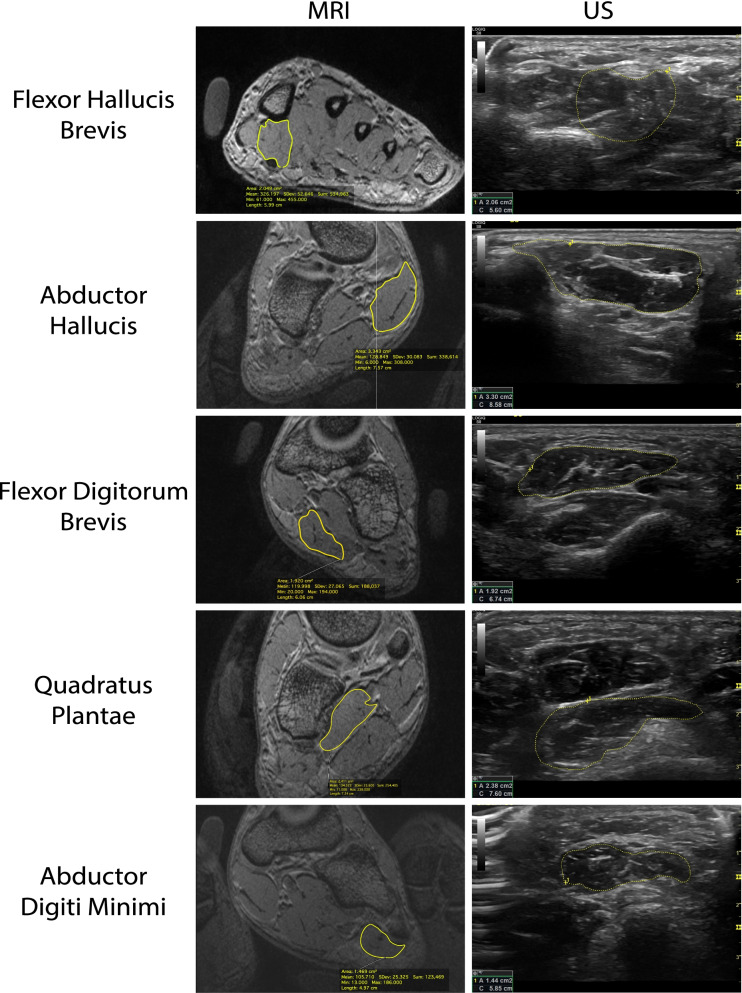
Intrinsic foot muscle images measured by MRI and US, corresponding muscles are highlighted via circumferential tracing

During imaging, participants were asked to contract specific muscles using toe flexion and toe spreading movements. Cine loops recorded the isolated contractions to help identify the muscle’s fascial borders and highlight any conformational muscle shape changes [34]. Each muscle was imaged in a relaxed state, in a contracted state, and then again in a relaxed state. All data were collected from the images in the relaxed state. Two separate recordings of the relax-contract-relax cycle were captured for each muscle. The transducer probe was briefly removed from the foot between recordings. Figure 4 presents a side-by-side comparison of muscle measurements via US and MRI.

### Data processing

Two researchers performed the image processing to obtain CSA muscle measurements for each muscle. One researcher performed all of the MR image processing, while another performed all of the US image measurements. These researchers did not compare results during the image processing, thus they were blinded to each other’s results.

 MRI scans for all 35 participants were loaded into Osirix (Pixmeo, Geneva, Switzerland) to determine the muscle’s CSA. CSA measurements were taken in the coronal plane from two adjacent slices for each of the five muscles assessed in this study in the foot as shown in Fig. 4 at the reference marks shown in Fig. 3, and averaged. Measurements were repeated twice consecutively for each of the five specific muscles within each slice for a total of two measurements per muscle as shown in Fig. 4. This procedure was repeated for the contralateral foot.

Ultrasound CSA measurements for all participants were obtained from two separate cine loop recordings for each of the five muscles for each foot. A single image was chosen from each cine loop while the muscle was at rest. Images of the muscle’s fascial border were captured and analyzed using software on the LOGIQ S8 machine via previously established methods [34, 35]. Muscles were imaged on the inside of the muscle fascia border. Measurements were repeated twice for each of the five specific muscles. See Fig. 4. One measurement was taken from the first cine loop and another measurement from the second cine loop. These two measurements were averaged and then compared to the average MR muscle measurements to determine inter-method validity. The two measurements were recorded separately to evaluate intratester reliability.

### Statistical analysis

Pearson product moment correlations were employed to determine whether the US system generated valid mean CSA results as compared with the MRI system. Intraclass-correlation coefficients (ICC_3,1_) were calculated to establish reliability using CSA measurements from each MRI and US image. To assess image segmentation repeatability, we chose the ICC model with fixed raters and random subjects. To identify testing error inherent to each imaging modality, we calculated the standard error of the measurement (SEm), a 95% confidence interval, and the minimum detectable difference (MDD) for both MRI and US using the following equations:

$$\mathrm{SEm}=\mathrm{SD}\;\left(\mathrm{Sq}\;\mathrm{rt}\;1-\;{\mathrm r}_{\mathrm{ICC}}\right)$$

$$95\%\;\mathrm{CI}\;\mathrm{SEm}\;=\;\mathrm{muscle}\;\mathrm{mean}\;\pm\;\left(1.96\;\ast\;\mathrm{SEm}\right)$$

$$\mathrm{MDD}\;=\;\mathrm{SEm}\;\ast\;1.96\;\ast\;\surd2$$

Bland-Altman plots were also generated to graphically highlight CSA difference and mean CSA between the MRI and US data, and to visualize any potential systematic error pattern or trends. The x-axis (mean CSA) on the Bland-Alman plots represents the average CSA from the MRI and US data (mean CSA = [MRI CSA + US CSA] ÷ 2) for each participant (*n* = 35). The y-axis (CSA difference) on the Bland-Alman plots represent the absolute difference between the CSA values (CSA difference = MRI CAS - US CSA) for each participant. We then calculated the percent muscle size based on the limits of agreement to help understand the variation between US and MRI derived CSA. Statistical analyses were performed using SPSS version 27.0 statistical software (IBM Corporation, Armonk, NY). An alpha of 0.05 was employed to determine statistical significance.

## Results

### Inter-method comparisons

**Fig. 5:**
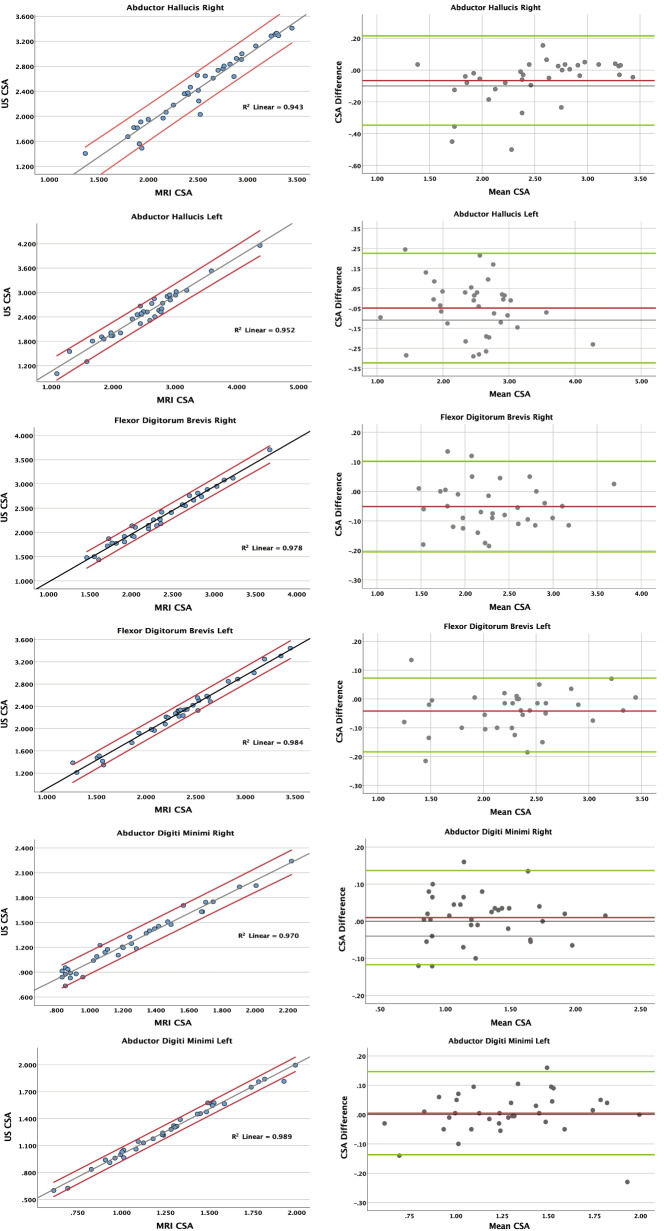
Correlational graphs and Bland-Altman plots – abductor hallucis, flexor digitorum brevis, abductor digiti minimi. Values are in cm^2^ (*n* = 35)

**Fig. 6:**
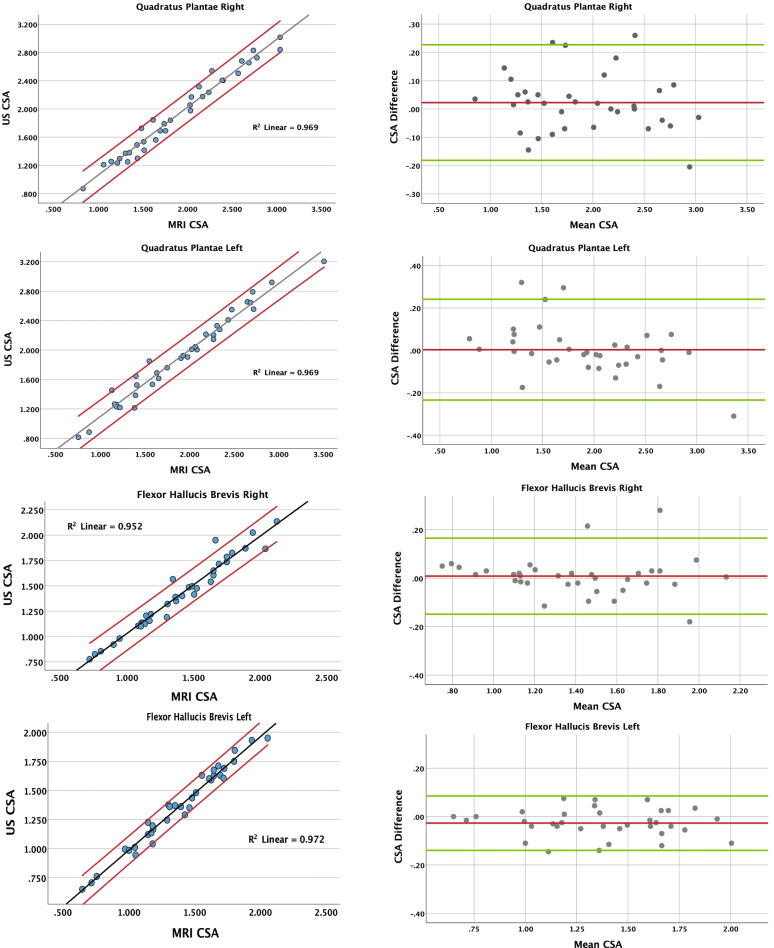
Correlational graphs and Bland-Altman plots – quadratus plantae, flexor hallucis brevis. Values are in cm^2^ (*n* = 35)

There were no significant differences between MR and US CSA muscles in any of the muscles assessed in the study (*p* > .05). High correlations (*r* = .971 to *r* = .995) were computed between the US and MRI mean CSA data (Table 1), while the Bland-Altman plots indicate no systematic error pattern (Figs. 5 and 6). Bland-Altman limits of agreement for percent muscle size were calculated ranging between 6 and 11% of muscle size (Table 1). Inter-method ICC values ranged from 0.960 to 0.993), showing excellent agreement between measurement methods.

**Table 1:** Mean muscle CSA, correlational coefficient values and Bland-Altman limits of agreement for US and MRI

Muscle	US	MRI	Inter-method ICC	r^*^	LoA Lower	LoA Upper	LoA % Average
Right foot							
FHB	1.41 ± 0.36	1.40 ± 0.36	0.988 (0.976 - 0.994)	0.976	-0.15	0.17	11.3
ABDH	2.48 ± 0.57	2.53 ± 0.51	0.982 (0.965 - 0.991)	0.971	-0.35	0.21	11.3
FDB	2.29 ± 0.53	2.36 ± 0.52	0.981 (0.963 - 0.990)	0.989	-0.20	0.10	6.6
QP	1.92 ± 0.58	1.90 ± 0.59	0.992 (0.984 - 0.996)	0.985	-0.18	0.23	10.6
ADM	1.28 ± 0.37	1.27 ± 0.37	0.985 (0.971 - 0.993)	0.985	-0.12	0.14	10.1
Left foot							
FHB	1.36 ± 0.34	1.37 ± 0.35	0.993 (0.986 - 0.996)	0.986	-0.14	0.09	8.5
ABDH	2.46 ± 0.6	2.51 ± 0.63	0.987 (0.974 - 0.993)	0.976	-0.32	0.23	11.2
FDB	2.27 ± 0.56	2.32 ± 0.54	0.960 (0.922 - 0.980)	0.992	-0.18	0.08	5.7
QP	1.92 ± 0.58	1.91 ± 0.64	0.990 (0.981 - 0.995)	0.985	-0.23	0.24	12.2
ADM	1.29 ± 0.34	1.28 ± 0.33	0.983 (0.967 - 0.992)	0.995	-0.14	0.15	11.2

*CSA *cross-sectional area (cm^2^; mean ± SD), *r *Pearson product correlations, *FHB *flexor hallucis brevis, *ABDH *abductor hallucis, *FDB *flexor digitorum brevis, *QP *quadratus plantae, *ADM *abductor digiti minimi. ^*^All *r*-values significant at *p* < .0001

### Intra-method comparisons

Mean muscle CSA ICC, and SEm values are outlined in Table 2. The mean muscle CSA values for US range from 2.48 to 1.28 cm^2^, while the MRI values range from 2.53 to 1.28 cm^2^. The SEm values for US range from 0.026 to 0.044 cm^2^, while the MRI values range from 0.018 to 0.023 cm^2^. The ICC values for US range from 0.991 to 0.997, while the MRI values range from 0.997 to 0.999. The absolute and relative MDD specific values are summarized in Table 3, with US values ranging from 0.077 to 0.110 cm^2^ and 6.02 to 3.89%, respectively; and MRI values ranging from 0.049 to 0.060 cm^2^ and 2.36 to 4.19%, respectively.

**Table 2:** Mean ICC and SEm values for US and MRI

Muscle	US - ICC_3,1_ (95% CI)	MRI - ICC_3,1_ (95% CI)	US - SEm (95% CI)	MRI - SEm (95% CI)
Right foot				
FHB	0.994 (0.989, 0.997)	0.998 (0.995, 0.999)	0.028 (1.35, 1.46)	0.020 (1.37, 1.43)
ABDH	0.997 (0.994, 0.998)	0.998 (0.995, 0.999)	0.031 (2.41, 2.53)	0.023 (2.49, 2.58)
FDB	0.993 (0.985, 0.996)	0.998 (0.997, 0.999)	0.044 (2.2, 2.37)	0.023 (2.31, 2.4)
QP	0.997 (0.995, 0.999)	0.999 (0.997, 0.999)	0.032 (1.86, 1.98)	0.019 (1.86, 1.94)
ADM	0.994 (0.989, 0.997)	0.997 (0.994, 0.998)	0.029 (1.22, 1.33)	0.020 (1.23, 1.31
Left foot				
FHB	0.991 (0.982, 0.995)	0.997 (0.995, 0.999)	0.032 (1.29, 1.42)	0.020 (1.34, 1.41)
ABDH	0.996 (0.991, 0.998)	0.999 (0.998, 0.999)	0.038 (2.38, 2.53)	0.012 (2.47, 2.54)
FDB	0.996 (0.993, 0.998)	0.999 (0.997, 0.999)	0.035 (2.2, 2.33)	0.017 (2.28, 2.35)
QP	0.996 (0.993, 0.998)	0.999 (0.998, 1.00)	0.037 (1.84, 1.99)	0.020 (1.87, 1.95)
ADM	0.994 (0.989, 0.997)	0.997 (0.993, 0.998)	0.026 (1.24, 1.34)	0.018 (1.25, 1.32)

All values reported in cm^2^. *ICC *intraclass correlation coefficients, *SEm *standard error of the measurement, *FHB *flexor hallucis brevis, *ABDH *abductor hallucis, *FDB *flexor digitorum brevis, *QP *quadratus plantae, *ADM *abductor digiti minimi

**Table 3:** Absolute and relative MDD values for US and MRI

	US		MRI	
Muscle	Absolute MDD	Relative MDD %	Absolute MDD	Relative MDD %
FHB	0.084	6.02	0.049	3.54
ABDH	0.096	3.89	0.060	2.36
FDB	0.110	4.82	0.056	2.37
QP	0.095	4.95	0.054	2.83
ADM	0.077	5.95	0.054	4.19

Average MDD values derived from combined left and right foot data (cm^2^). Absolute minimum detectable difference (MDD) values calculated using MDD = SEm ∗ 1.96 ∗ √2. Relative MDD values calculated using MDD% = [MDD / CSA average] * 100

## Discussion

The results of this study support our hypotheses. The findings indicate that our US method can reliably quantify the intrinsic foot muscle CSA and that it has high agreement with MR based measurements. This is demonstrated by the high precision seen in the Bland-Altman plot analysis and a lack of statistical difference between the US and MR based measurements. This relationship is supported by a high Pearson product moment correlations and excellent inter-method ICC values. These values in aggregate establish the validity of US CSA measurements compared to MRI for intrinsic foot muscles. Our inter-method comparison of CSA supports the validity of US assessment of foot muscle CSA to that derived with MRI. Our high correlational results between MRI and US (*r* = .971 to 0.995) were expected and these are similar to other studies. For example, Kositsky et al. reported r-values between 0.882 and 0.996 when comparing MRI and US measurements involving the hamstring muscle and hamstring tendon. Similarly, Ahtiainen et al. [36] and Van et al. [37] reported r-values ranging from 0.91 to 0.98 when comparing MRI and US measurements involving the quadriceps and core trunk muscles, respectively. Additionally, the inter-method ICC ranging from 0.960 to 0.993 suggest excellent agreement between measurement methods. Our Bland-Altman plots further support the high agreement and low error rates between MRI and US. Bland-Altman percent muscle size limits of agreement from our study range from 6– to 2%. This indicates that US measurements possess a high level of precision and that we can expect a potential error of up to 6–12% depending on the muscle, when measuring intrinsic foot muscle size with US compared to MRI. Our limits of agreement values compare favorably with studies investigating reliability of US measurements of intrinsic foot muscle CSA that reported values ranging from 8 to 30% [25], from 4.6 to 9.7% [35], from 11 to 26% [26], and from 3.7 to 11.5% [34]. The Bland-Altman plots do not exhibit any proportional bias, as indicated by lack of sloping trend lines. In addition most of the plots show a small negative bias, showing that US tended to slightly under-estimate the muscle sizes compared to MRI. However, since the bias is very small (ranging between 0.06 and 0.0), it is supportive of a high degree of accuracy between these measurements [38]. The analyses derived from the Bland-Altman plots show high agreement between measurement methods and therefore the validity of US assessment of foot muscle CSA.

Our intra-method ICC data (0.991 to 0.999) demonstrate excellent repeatability within both ultrasound and MRI methods. Our ICC values are equal to or slightly higher than in other studies (0.89 to 0.99) that employed US to measure intrinsic foot muscle CSA [25, 26]. The most likely reasons for some of our slightly higher reliability results was the our use of cine loops [34]and the fact that we averaged two separate US measurements and collected these trials during the same data collection session [39].

The MDD provides further insight into the utility of a measurement by establishing the expected measurement error. The MDD is the smallest difference or change that can be detected indicating a threshold for deciding that a real change has occurred [40]. A lower MDD indicates less error in the measurement method. Our MDD results suggest that the MRI and US protocols can detect a muscle CSA change of about 0.05 cm^2^ and 0.09 cm^2^, respectively (Table 3). Similarly, Del-Baño Aledo [41] found an average MDD of 0.03 to 0.07 cm2 when evaluating the Achilles tendon, patellar tendon and elbow common extensor tendon with US. In terms of relative MDD values, the MRI is able to detect a muscle size change of 2.4–4.2%, while US can detect a muscle size change of 3.9–6.0% (Table 3). These values are similar to those reported in two previous MRI studies evaluating the quadriceps femoris CSA (2.2–4.4%) and calf muscle CSA (3.0–3.4%) [42, 43]. Small MDD values are important when monitoring patients before, during, or after an exercise program to know that changes in muscle size are most likely due to actual muscle size changes and not because of spurious measurement error.

Importantly, our results demonstrate that US is a valid modality to determine intrinsic foot muscle CSA. These results are both statistically significant and clinically significant. Statistically our results exceed a confidence level of 99%, or an approximate 1% probability that our findings are due to random chance (Table 1). Our results are significant since they indicate US measurements are accurate and closely match the corresponding MRI data. Consequently, clinicians and researchers can choose to use US if they wish, with the confidence that the intrinsic foot muscle CSA measurements will be comparable to MRI. This is especially meaningful since the US equipment and methodology is (1) widely available, (2) relatively inexpensive, (3) time-efficient, (4) safe, (5) able to show individual real-time muscle activation through cine loops [34], and (6) able to provide immediate muscle CSA results and post-measurement exercise training recommendations [44]. Nevertheless, our findings support the MRI protocol as the criterion standard, since it provides slightly more consistent results than US, with lower SEm and MDD values when measuring intrinsic foot muscle CSA.

While our study was strengthened by the number of participants completing the study, it also had potential limitations. First, we only recruited young adult and middle-age participants that suggests that our results cannot be generalized to other age groups. First, we only recruited young-adult and middle-age participants with no pathological conditions, limiting external generalization to this population. Second, we did not measure the interossei, lumbricals, flexor digiti minimi brevis nor the intrinsic extensor muscles. While these muscles may play important roles in foot function, they present with different challenges to imaging. Future research could focus on developing techniques to image and measure these muscles in isolation. Consequently, our MRI and US accuracy comparisons could have been slightly different had we chosen to measure other or all intrinsic foot muscles.

## Conclusions

Although US offers many practical advantages, our results confirm MRI as the criterion measure with higher precision and reliability compared to US when measuring intrinsic foot muscle CSA. In comparison to MRI, our results suggest that US yields relatively accurate and reliable intrinsic foot muscle CSA measurements, making it a valid and viable alternative to MRI in research and clinical settings.

## Data Availability

The datasets generated and/or analyzed during the current study are not publicly available as approval was not granted by the ethical review board nor was a statement included in the informed consent that study data would be deposited in a research depository. However, researchers seeking access to de-identified data should make a reasonable request to the corresponding author and an appeal to the Human Research Protection Program and Institutional Review Board can be sought.

## Abbreviations

US: Ultrasound
MRI: Magnetic Resonance Imaging
FHB: Flexor hallucis brevis
ABDH: Abductor hallucis
FDB: Flexor digitorum brevis
QP: Quadratus plantae
ADM: Abductor digiti minimi
CSA: Cross Sectional Area
ICC: Intraclass Correlation Coefficient
SEm: Standard Error of the Measurement
MDD: Minimal Detectable Difference

## Declarations

All experimental protocols were approved by Brigham Young University’s Institutional Review Board of the Human Research Protection Program, study protocol, IRB2019-375). Informed consent was obtained from each study participant who participated in this study in accordance with the relevant guidelines and regulations to meet appropriate safeguards to protect the rights and welfare of research subjects according to the Human Research Protection Program and its Institutional Review Board at Brigham Young University. The study was assigned study number, IRB2019–375. All study participants were 18 years or older and able to provide their own informed consent.
